# Combined Fluorescence-Based in Vitro Assay for the Simultaneous Detection of Cell Viability and Alkaline Phosphatase Activity during Osteogenic Differentiation of Osteoblast Precursor Cells

**DOI:** 10.3390/mps3020030

**Published:** 2020-04-26

**Authors:** Sebastian Wilkesmann, Fabian Westhauser, Joerg Fellenberg

**Affiliations:** Center for Orthopedics, Trauma Surgery and Paraplegiology, University of Heidelberg, 69118 Heidelberg, Germany

**Keywords:** osteogenic differentiation, alkaline phosphatase, human mesenchymal stromal cell, osteoblast precursor cell

## Abstract

Novel bone substitute materials need to be evaluated in terms of their osteogenic differentiation capacity and possible unwanted cytotoxic effects in order to identify promising candidates for the therapy of bone defects. The activity of alkaline phosphatase (ALP) is frequently quantified as an osteogenic marker, while various colorimetric assays, like MTT assay, are used to monitor cell viability. In addition, the DNA or protein content of the samples needs to be quantified for normalization purposes. As this approach is time consuming and often requires the analysis of multiple samples, we aimed to simplify this process and established a protocol for the combined fluorescence-based quantification of ALP activity and cell viability within one single measurement. We demonstrate that the fluorogenic substrate 4-methylumbelliferone-phosphate (4-MUP) and the commonly used para-nitrophenylphosphate (p-NPP) produce comparable and highly correlating results. We further show that fluorescein–diacetate (FDA) can be used to quantify both cell viability and cell number without interfering with the quantification of ALP activity. The measurement of additional normalization parameters is, therefore, unnecessary. Therefore, the presented assay allows for a time-efficient, simple and reliable analysis of both ALP activity and cell viability from one sample and might facilitate experiments evaluating the osteogenic differentiation of osteoblast precursor cells.

## 1. Introduction

Many bone substitute materials, e.g., bioactive glasses, are known to promote osteogenic differentiation in osteoblast precursor cells like human mesenchymal stromal cells, making them therapeutic options for the repair of bone defects [1]. However, novel upcoming materials need to be evaluated in terms of their osteogenic differentiation and cytotoxicity in order to identify promising approaches.

Besides the quantification of extracellular calcium deposits or the gene expression analysis of osteogenic marker genes, alkaline phosphatase (ALP) activity functions as an approved marker for osteogenic differentiation of in vitro cultured osteoblast precursor cells [2,3]. ALP activity is commonly determined by the absorbance-based para-nitrophenylphosphate (p-NPP) assay [4,5,6]. ALP converts the transparent p-NPP into the yellow para-nitrophenol (p-NP). This color change can be quantified by photometry. As the absolute ALP activity of a sample lacks explanatory power, values usually need to be normalized to the total number of cells or correlating values in a second step. Comparing the ALP activity to the total protein or dsDNA concentration of the investigated sample are approved ways to give reasonable context to the obtained data [6,7]. 

ALP is known to be an early marker of osteogenic differentiation on a cellular level, thus correlating with the activity and presence of osteoblasts that develop from bone precursor cells during differentiation [2,8]. However, ALP is also expressed in other cell types, e.g., pluripotent stem cells [9]. Other assays can be used to analyze the development of the extracellular matrix—for example, Alizarin Red staining, which detects calcium deposition in later stages of differentiation [10]. Although Alizarin Red staining might be the more robust marker, it is not applicable in many experimental settings, including the one used in this study: bioactive glasses are characterized by their intrinsic ability to precipitate a carbonate-substituted hydroxyapatite layer upon contact with physiological fluids. This layer would also be detected by the Alizarin Red staining without being a direct marker of osteogenic differentiation and the formation of a calcified extracellular matrix [11]. In context with bioactive glass ceramic granules (BGs) the Alizarin Red staining assay is mostly used in indirect culture settings (e.g., during the introduction of ionic dissolution products to the cell culture) and not in direct culture settings where cells are co-cultured with BGs [12,13]. We therefore focused on the more versatile quantification of ALP activity.

In addition, cell viability is an important parameter that must be considered during the evaluation of novel materials for the treatment of bone defects. Several established assays are frequently used for this purpose, including the colorimetric MTT, MTS and water-soluble tetrazolium (WST) assays, as well as fluorometric assays like calcein-AM and fluorescein–diacetate (FDA) staining [14,15,16,17].

As the individual measurement of alkaline phosphatase activity, cell viability and normalization parameters can be very time consuming, especially when multiple materials and cell types need to be investigated, we aimed to simplify these measurements by a combined fluorescence-based approach.

As a substrate for ALP, we used 4-methylumbelliferone-phosphate (4-MUP). ALP converts non-fluorescent 4-MUP into blue-fluorescent 4-methylumbelliferone (4-MU) [18]. A substrate with blue fluorescence was selected to avoid interference with the green fluorescent fluorescein that is generated during the analysis of cell viability using FDA. The cell membrane passing FDA is converted by the intracellular esterases of living cells to fluorescein, while the intensity of the emerging green fluorescent signal correlates with the viability and number of cells in the tested sample [19]. Finally, the total protein concentration of the investigated cells can be evaluated from the same sample using commercially available absorbance-based protein assays, like the bicinchoninic acid (BCA)-based assay, providing an additional option for ALP normalization.

The major advantage of this approach is that, in contrast to common practice, the two important biological parameters of osteogenic differentiation and viability, instead of either one of those, can be assessed in one experiment. Additionally, this sensitive fluorescence-based approach can be performed in a 96-well culture plate setting from start to end, making it possible to test multiple replicates and conditions easier and faster. This protocol is primarily intended for the analysis of in vitro experiments. However, if appropriate tissue digestion protocols are available, this method can also be used with three-dimensional in vitro or even in vivo samples [6]. If only ALP activity is of interest, the sample may be homogenized in lysis buffer without digestion and directly used for ALP activity measurement.

## 2. Experimental Design

**Figure 1:**
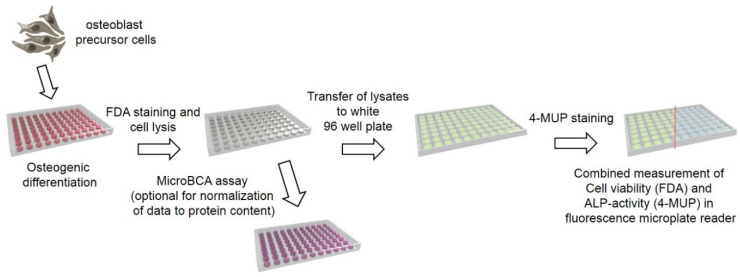
Schematic overview of the protocol.

As the combined assay aims to determine cell viability, osteogenic differentiation and total protein content, the assay can be subdivided into two parts, before and after cell lysis. In the first steps, the FDA staining requires viable cells, whose esterases convert the non-fluorescent FDA into the fluorescent fluorescein. After washing and cell lysis, fluorescein and ALP are released, and ALP activity can be determined in the same lysate by the conversion of non-fluorescent 4-MUP into fluorescent 4-MU. Finally, both emerging products are quantified using a microplate fluorescence reader. As an additional normalization parameter, the total amount of protein can be assessed in the same lysate using a MicroBCA kit (Thermo Fisher, Dreieich, Germany) In this case, an absorbance-based assay is used. A schematic overview of the assay is presented in Figure 1. All obtained fluorescence intensities and absorbance values can be interpreted relative to an untreated control. If absolute quantification is desired, standards for the ALP activity and total protein concentration can be prepared.

### 2.1. Materials

- 1.5x ALP Assay Buffer (75 mM Tris pH 9.3, 1.5 mM MgCl_2_, 0.15 mM ZnCl_2_ (all Carl Roth));
- ALP standard working solution (0.4 mU/µL) (shrimp alkaline phosphatase (Thermo Fisher));
- Phosphate-buffered saline (PBS) (Thermo Fisher);
- FDA (Thermo Fisher) working solution (2 µg/mL in PBS);
- 0.5% Triton-X-100 (Sigma-Aldrich, Steinheim, Germany) in PBS (Thermo Fisher) 4-Methylumbelliferyl Phosphate (4-MUP) (Thermo Fisher) working solution (100 µM in 1.5x ALP assay buffer);
- Distilled water for buffer preparation;
- MicroBCA kit (Thermo Fisher) (optional);
- Protein standard working solution (400 µg/mL) (optional, included in the MicroBCA kit).

### 2.2. Equipment

- 96-well cell culture plates transparent (Sarstedt, Nümbrecht, Germany) 96-well cell culture plates black or white (Kisker Biotech GmbH & Co. KG, Steinfurt, Germany)
- Fluorescence microplate reader (Wallac 1420 Victor 2, Perkin Elmer, Waltham, MA) Filter: 485/530 (ex/em) and 360/440 (ex/em)

### 2.3. Study Ethics 

For the isolation of the primary cells used in this study, informed consent was obtained from all individuals prior to harvesting. The study has been approved by the authors’ institutional review board (S-340/2018). 

### 2.4. Primary Cells and Cell Lines 

For the evaluation of the developed assay, several cell types were used, including primary human mesenchymal stromal cells (hMSCs), human osteoblasts (hOBs) and osteosarcoma cell lines. The isolation of hMSCs was achieved from bone marrow washouts as previously described [5,20,21]. In brief, cells were fractionated on a Ficoll Paque Plus density gradient (GE Healthcare Europe, Freiburg, Germany), washed in PBS (Thermo Fisher) and cultured in flasks coated with 0.1% gelatin (Sigma-Aldrich). Culture medium consisted of Dulbecco’s modified Eagle medium (DMEM) high glucose supplemented with 12.5% fetal calf serum (FCS), 2 mM L-glutamine, 1% non-essential amino acids (NEAA), 50 μM β-mercaptoethanol (all Thermo Fisher), 100 μg/mL penicillin/streptomycin (Biochrom, Berlin, Germany) and 4 ng/mL fibroblast growth factor 2 (Abcam, Berlin, Germany). Non-adherent cells were removed after 24 h of cultivation and adherent cells were passaged at 80% confluency.

hOBs were isolated from pieces of cancellous bone that were cut into small fragments and digested in DMEM supplemented with 1 mg/mL Collagenase Type II (Thermo Fisher) for 2 h at 37 °C under continuous rotation. After the digestion period, bone fragments and cells were washed twice in PBS and cultured as described for hMSCs. At 80% confluency, cells were passaged, and remaining bone fragments were discarded.

The following osteosarcoma cell lines were purchased: Saos-2 (CLS Cell Lines Service, Eppelheim, Germany, #300331), HOS (ATCC^®^ CRL-1543), MG-63 (ATCC^®^ CRL-1427) and U2OS (CLS Cell Lines Service, #300364).

## 3. Procedure

1. FDA staining (10 min)
  - Culture cells in 96-well plates (5000 cells/well)
  - Treat cells as desired
  - Discard media from the 96-well cell culture plate
  - Wash cells once with 100 µL PBS
  - Add 100 µL of FDA working solution
  - Incubate for 5 min at 37 °C
  - Discard FDA working solution
  - Wash cells once with 100 µL PBS
2. Cell lysis (5 min)
  - After the last wash, discard PBS and add 150 µL of 0.5% Triton-X-100 in PBS
  - Incubate for 5 min at 37 °C
3. Preparation of ALP and Protein standard (10 min)
  (1) ALP standard
    - Add 50 µL of distilled H_2_O to wells A1 to H1 and A2 to H2 of a black or white 96-well cell culture plate
    - Add 50 µL of ALP standard working solution to wells A1 and A2
    - Transfer 50 µL of diluted ALP-standard from A1 to B1, from B1 to C1, from C1 to D1, from D1 to E1, from E1 to F1, from F1 to G1 and discard 50 µL from G1. The resulting standards range from 10 mU to 0.16 mU ALP
    - Repeat the previous two steps for wells A2 to H2
    - H1 and H2 represent the blanks
  (2) Protein standard (optional)
    - Add 75 µL of distilled H_2_O to wells A1 to H1 and A2 to H2 of a transparent 96-well cell culture plate
    - Add 75 µL of protein standard working solution to well A1 and A2
    - Transfer 75 µL of diluted ALP-standard from A1 to B1, from B1 to C1, from C1 to D1, from D1 to E1, from E1 to F1, from F1 to G1 and discard 75 µL from G1. The resulting standards range from 200 µg/mL to 3.125 µg/mL BSA
    - Repeat the previous two steps for wells A2 to H2
    - H1 and H2 represent the blanks
4. 4-MUP staining and fluorescence quantification (25 min)
  - Mix cell lysates by pipetting up and down several times and transfer 50 µL of lysate into the empty wells of the white 96-well cell culture plate with the ALP standards
    ○
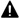
**CRITICAL STEP:** Mix and transfer gently to avoid foam formation
  - Add 100 µL of 4-MUP working solution to all wells, including the standards and blanks
  - Incubate for 20 min at 37 °C
  - Quantify the fluorescence intensity of fluorescein at 485/535 (ex/em) with a microplate fluorescence reader
  - Quantify the fluorescence intensity of 4’MU at 360/440 (ex/em) with a microplate fluorescence reader
5. Total Protein measurement (optional) (2 h and 10 min)
  - Transfer 75 µL of cell lysates into the empty wells of the prepared transparent 96-well cell culture plate containing the protein standard
    ○
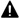
**CRITICAL STEP:** Transfer gently to avoid foam formation
  - Add 75 µL of Micro BCA working solution to all wells, including the standards and blanks
  - Incubate for 2 h at 37 °C
  - Cool the plate to room temperature (RT)
  - Quantify the absorption in a microplate reader at ex 562 nm

Total time for completion: 3 h 

## 4. Expected Results

### 4.1. The Fluorescence-Based 4-MUP and Absorbance-Based p-NPP Assays Produce Comparable Results

In order to validate that the 4-MUP assay specifically measures ALP activity, eight ALP-standard samples ranging from 0.16 mU to 10 mU of shrimp alkaline phosphatase (Thermo Fisher) were prepared. ALP activity was determined using 4-MUP, as well as the conventional p-NPP as substrate. The p-NPP assay was performed following a previously described protocol [5], the 4-MUP assay was performed according to the provided protocol, neglecting the steps concerning the FDA staining as only ALP activity was measured. The measurements were performed in triplicate. Additionally, the ALP activity in 68 samples of six different cell types, including hMSCs, hOBs and the osteoblast-like osteosarcoma cell lines MG-63, HOS, U2OS and Saos-2 was quantified using 4-MUP and p-NPP as substrates, as described above. The cells were co-cultured with 45S5 bioactive glass ceramic granules (BG) in different concentrations to induce osteogenic differentiation [22]. A rapid BG-induced increase in ALP activity has already been shown in previous studies [23]. Briefly, cells were seeded in a density of 1.84 × 10^4^ cells/cm^2^ in a 24-well plate (Thermo Fisher) and incubated in BG-supplemented and BG-free cell culture media, consisting of DMEM supplemented with 10% fetal calf serum and 1% penicillin/streptomycin (all Thermo Fisher) for up to 7 days. After washing with PBS, cells were lyzed using 500 µL 0.5% Triton-X-100 in PBS and stored at −80 °C until both assays were performed. The measurements were performed in technical duplicates.

**Figure 2:**
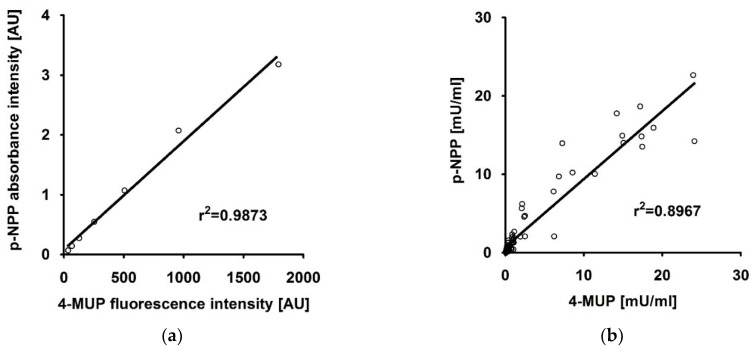
(**a**) Alkaline phosphatase (ALP) activity of ALP-standards (0.16 mU to 10 mU) quantified using 4-methylumbelliferone-phosphate (4-MUP) (x-axis) or para-nitrophenylphosphate (p-NPP) (y-axis) as substrate. The continuous line shows the linear regression curve with r^2^ = 0.9873. (**b**) ALP activity data from 68 samples obtained with 4-MUP (x-axis) and p-NPP (y-axis) as substrate. The continuous line shows the linear regression curve with r^2^ = 0.8967.

As shown in Figure 2a,b the results of the two compared ALP activity measurement protocols showed a high correlation when applied for the quantification of ALP-standards (r^2^ = 0.9873) and cell lysates (r^2^ = 0.8967), respectively, with r corresponding to the Pearson correlation coefficient.

### 4.2. FDA Fluorescence Intensity Correlates With the Number of Viable Cells

For the interpretation of measured ALP activity, the obtained data are commonly normalized to the total number of cells, total amount of dsDNA, or total amount of protein in the sample [3,7]. In the presented combined approach, FDA fluorescence intensity quantifies the viability of the cells in the investigated sample. In order to investigate whether the determined cell viability correlates with the total number of cells and might thus be used for the normalization of ALP activity data, increasing amounts of HOS cells were seeded in 96-well plates. Cell numbers were quantified by flow cytometry (MACS Quant, Miltenyi Biotech, Bergisch Gladbach, Germany) before seeding. After cell attachment, the combined 4-MUP/FDA assay was performed as presented. Each measurement was performed in triplicate.

**Figure 3:**
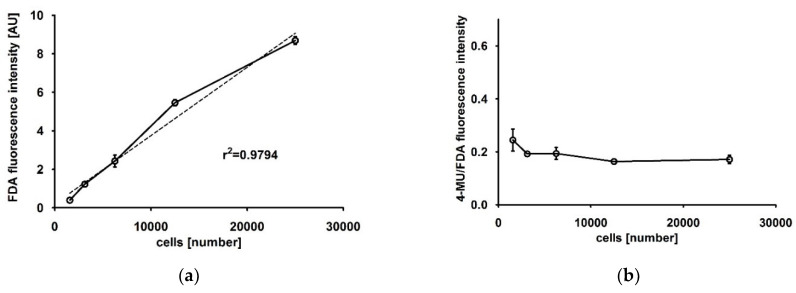
(**a**) Fluorescein–diacetate (FDA) fluorescence intensity for 1563, 3125, 6250, 12,500 and 25,000 HOS cells. The dotted line indicates the linear regression curve with r^2^ = 0.9794. (**b**) 4-methylumbelliferone (4-MU)/FDA fluorescence intensity ratios calculated from 1563, 3125, 6250, 12,500 and 25,000 HOS cells.

The resulting viability values showed a high correlation (r^2^ = 0.9794) with the number of seeded cells (Figure 3a), suggesting that the obtained FDA fluorescence intensities might be directly used for the normalization of ALP activity data. As a consequence, the 4-MUP/FDA ratios are stable over a wide range of measured cell numbers (Figure 3b).

### 4.3. FDA and 4-MUP Fluorescence Signals Do not Interfere in the Combined Approach

**Figure 4:**
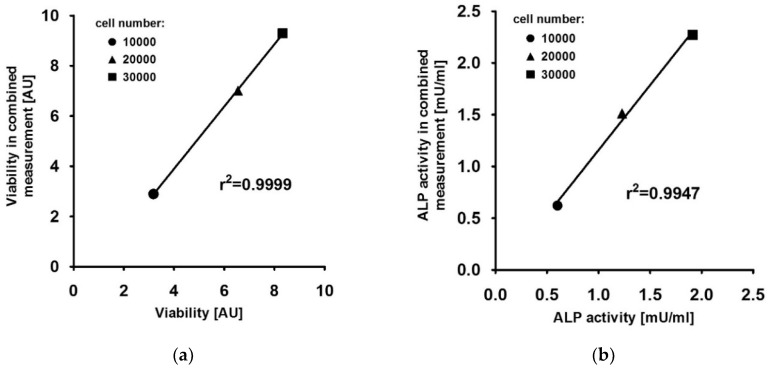
(**a**) Viability of HOS cells determined by an individual FDA staining (x-axis) or in combination with an ALP (4-MUP) measurement (y-axis). The continuous line shows the linear regression curve with r^2^ = 0.9999. (**b**) ALP activity of HOS cells determined by an individual 4-MUP staining (x-axis) or in combination with a viability (FDA) measurement (y-axis). The continuous line shows the linear regression curve with r^2^ = 0.9947.

FDA fluorescence intensity is quantified at 485/535 nm (ex/em), 4-MU fluorescence intensity is quantified at 360/440 nm (ex/em). Therefore, the two signals should not interfere and both measurements should not affect each other. To prove this, 10,000, 20,000 and 30,000 cells were seeded in 96-well plates. Once the cells were attached, the following assays were performed in triplicate for each cell number: the combined viability and ALP measurement as presented, an exclusive viability measurement using FDA as a substrate and an exclusive ALP measurement using 4-MUP as a substrate. These exclusive approaches were performed according to the presented protocol, omitting either FDA or 4-MUP as a substrate. The mean value of the obtained results of the combined approach was related to the mean value of the individual experiments. As seen in Figure 4, there is a high correlation between the combined and exclusive measurements, with r^2^ = 0.9999 and r^2^ = 0.9947 for viability and ALP activity, indicating that both measurements do not interfere with each other when applied within the same sample. 

## 5. Reagents Setup

1. ALP Standard
  - Stock solution: 1 U/µL Shrimp alkaline phosphatase (Thermo Fisher)
  - Working solution: ALP stock solution diluted 1:2500 in distilled H_2_O (400 µU/µL)
2. Protein Standard
  - Stock solution: 2 mg/mL Albumin standard (Thermo Fisher)
  - Working solution: Albumin standard stock solution diluted 1:5 in distilled H_2_O (400 µg/mL)
3. 10x ALP Assay Buffer (storage at 4 °C)
  - 500 mM TRIS pH 9.3 (Carl Roth, Karlsruhe, Germany)
  - 10 mM MgCl_2_ (Carl Roth)
  - 1 mM ZnCl_2_ (Carl Roth)
4. FDA substrate solution
  - Stock solution: 1 mg/mL FDA (Thermo Fisher) in acetone (Carl Roth) (storage at −20 °C)
  - Working solution: FDA stock solution diluted 1:50 in PBS (20 µg/mL)
5. 4-MUP substrate solution
  - Stock solution: 5 mM 4-MUP (Thermo Fisher) (storage at 4 °C)
  - Working solution: 4-MUP Stock solution diluted 1:50 in 1.5x ALP Assay Buffer (100 µM)
6. Micro BCA substrate solution
  - Micro BCA™ Protein Assay Kit: mix according to manufacturer’s instruction (Thermo Fisher)
